# Core Psychiatry Training in Practice: A Cross-Sectional Evaluation of Trainee Experience Within Core Psychiatry Rotation

**DOI:** 10.1192/bjo.2026.11347

**Published:** 2026-06-30

**Authors:** Esther Anekwu, Joy Owens

**Affiliations:** 1 Sheffield Health Partnership University NHS Foundation Trust, Sheffield, United Kingdom; 2 Sheffield Children's NHS Foundation Trust, Sheffield, United Kingdom

## Abstract

**Aims:**

Core Psychiatry Training is designed to provide structured clinical experience, supervision, and professional development while supporting trainee wellbeing and progression. However, the lived experience of trainees within training programmes, particularly in relation to supervision, workload, assessment burden, and support for examinations, is less frequently explored in depth. Understanding these experiences is important for maintaining training quality and supporting trainee confidence and retention.

This study explores the experiences of doctors enrolled in Core Psychiatry Training across core Psychiatry rotation, focusing on the quality of supervision, workload pressures, assessment burden, well-being support, and perceived preparedness for examinations and progression.

**Methods:**

An anonymous cross-sectional survey was distributed to all 47 Core Psychiatry Trainees (CT1–CT3) within the South Yorkshire Rotation. The questionnaire covered key aspects of training, including induction, clinical and educational supervision, teaching access, workload and working patterns, workplace-based assessments and portfolio requirements, ARCP experience, wellbeing support, inclusion, and career development. Responses were analysed using both descriptive statistics and thematic synthesis to identify patterns and tensions across placements. This study did not meet the NIHR “What is Research” criteria and therefore did not require NHS Research Ethics Committee review.

**Results:**

Thirty of the 47 eligible trainees responded. Trainees’ experiences wereheterogeneous and strongly placement dependent. Supervision was generally valued when accessible, particularly for clinical decision-making and risk management; however, the frequency, consistency, and scope of supervisory support varied considerably. Educational supervision was more commonly experienced as administrative than developmentally proactive, with limited structured support for examination preparation or career planning.

Workload pressures were frequently reported, with many trainees working beyond contracted hours and experiencing difficulty taking breaks. Portfolio and assessment requirements were widely viewed as time-consuming, contributing to stress around ARCP preparation despite general perceptions of procedural fairness. Support for examination preparation was often informal or self-directed, influencing trainee confidence and increasing reliance on peers rather than supervisors.

Awareness of wellbeing support varied, and feeling supported did not always correlate with knowledge of available resources. While most trainees reported feeling respected within their teams, some lacked confidence in seeking help around exams or performance concerns.

**Conclusion:**

This evaluation highlights substantial variation in the lived experiences of Core Psychiatry Trainees within core training Rotation. While trainees value supervision and support when available, inconsistencies in engagement, exam-focused guidance, workload protection, and wellbeing support impact confidence, stress, and perceived preparedness for progression. Capturing trainee experience in this way provides an important foundation for reflective review of training delivery and trainee support.

